# Association between systemic immune-inflammation index and metabolic syndrome and its components: results from the National Health and Nutrition Examination Survey 2011–2016

**DOI:** 10.1186/s12967-023-04491-y

**Published:** 2023-10-04

**Authors:** Yang Zhao, Wenyu Shao, Qihan Zhu, Rui Zhang, Tao Sun, Bijia Wang, Xiaofei Hu

**Affiliations:** 1 Department of Nuclear Medicine, Southwest Hospital, Third Military Medical University (Army Medical University), Chongqing, China; 2 Department of Basic Medicine, Third Military Medical University (Army Medical University), Chongqing, China; 3 Department of Biomedical Engineering, Fourth Military Medical University, Xi’an, China; 4 Department of Neurology, Southwest Hospital, Third Military Medical University (Army Medical University), Chongqing, China

**Keywords:** NHANES, Systemic immune-inflammation index, Metabolic syndrome

## Abstract

**Background:**

Metabolic syndrome (MetS), a worldwide public health problem, affects human health and quality of life in a dramatic manner. A growing evidence base suggests that MetS is strongly associated with levels of systemic immune inflammation. The present study aimed to investigate the possible relationship between the systemic immune-inflammation index (SII), a novel inflammatory marker, and MetS to provide data support for effective MetS prevention by reducing the systemic inflammatory response.

**Methods:**

We included adult participants with complete SII and MetS information from the 2011–2016 National Health and Nutrition Examination Survey (NHANES). MetS was defined as using the criteria developed by the Adult Treatment Program III of the National Cholesterol Education Program. The formula for SII was as follows: SII = platelet counts × neutrophil counts/ lymphocyte counts. Weighted linear regression was used to assess differences in variables across SII quartile groups after the SII score was divided into 4 quartiles. The independent interaction between SII and MetS was investigated using weighted multivariate logistic regression analysis and subgroup analysis, and the relationship between SII levels and 5 particular MetS items was further explored in depth.

**Results:**

A total of 12,402 participants, 3,489 of whom were diagnosed with MetS, were included in this study. After correcting for covariates, the results of a logistic regression of multistage weighted complex sampling data revealed that participants with higher SII scores had a higher chance of developing MetS (odds ratio (OR) = 1.33, 95% confidence interval (CI): 1.14–1.55) and that SII levels could be used as an independent risk factor to predict that likelihood of MetS onset. In the Q1–Q4 SII quartile group, the risk of developing MetS was 1.33 times higher in the Q4 group, which had the highest level of systemic immune inflammation than in the Q1 group. After adjusting for all confounding factors, SII scores were found to have a negative correlation with high-density lipoprotein cholesterol (OR = 1.29; 95% CI, 0.99–1.67, P = 0.056) and a significant positive correlation with waist circumference (OR = 2.17; 95% CI, 1.65–2.87, P < 0.001) and blood pressure (BP) (OR = 1.65; 95% CI, 1.20–2.27, P = 0.003). Gender, age, and smoking status were shown to alter the positive association between SII and MetS in subgroup analyses and interaction tests (*p* for interaction < 0.05). Additionally, we demonstrated a nonlinear correlation between SII and MetS. The findings of the restricted cubic spline indicated that there was an inverted U-shaped association between SII and MetS.

**Conclusions:**

Our findings imply that increased SII levels are related to MetS, and SII may be a simple and cost-effective method to identify individuals with MetS. Therefore, protective measures such as early investigation and anti-inflammatory interventions are necessary to reduce the overall incidence of MetS.

## Introduction

According to the National Cholesterol Education Program-Adult Treatment Panel III criteria, metabolic syndrome (MetS) is a group of metabolic disorders that includes elevated fasting glucose, hypertension, obesity, elevated triglycerides (TG), and decreased high-density lipoprotein cholesterol (HDL-C) [1]. According to statistics, the prevalence of MetS in Americans has increased from 25.29% in 1988 to 34.7% in 2016 [2]. MetS has therefore become a severe public health issue, with mounting evidence that it may be linked to an increased risk of coronary heart disease, cardiovascular disease (CVD), type 2 diabetes (T2D), stroke, and all-cause mortality [3]. Given the great danger posed by MetS to human health, how to detect and intervene early in MetS is currently a hot topic of discussion among scholars in related fields.

Numerous studies have demonstrated a notable correlation between MetS and both systemic inflammation and immune response. The prevailing perspective posits that MetS originates from excessive obesity [4], which is characterized by mild chronic inflammation. Inflammatory factors, secreted by adipose tissue, contribute to the development of metabolic disorders by instigating a systemic inflammatory response [5]. Additionally, abnormal triglyceride levels and hyperglycemia have been linked to inflammation [6]. Studies have reported that hyperglycemia diminishes the body's immune response to external infections through the suppression of inflammatory factors, including IFN-γ and TNF-α, which are secreted by T cells [7, 8].

What’s more, previous studies have indicated that immune cell activation plays a role in the development of T2D, obesity-associated insulin resistance, and other metabolic disorders [9–11]. Satoshi et al. found that CD8 + T cells can activate macrophages in adipose tissue [12], leading to an alteration in the immune microenvironment and a shift in adipose tissue from an anti-inflammatory to a pro-inflammatory state [13]. So the control of inflammation seems to be a crucial intervention for mitigating metabolic disorders. However, population-based studies on the association between inflammation and metabolic disorders remain limited. To comprehensively investigate this potential link, there is an immediate requirement for an objective evaluation metric that can accurately reflect the status of immunity and inflammation in the MetS population.

The systemic immune-inflammation index (SII), created by Hu et al. is a comprehensive new immunoinflammatory biomarker based on platelet, neutrophil, and lymphocyte counts that accurately reflect the body’s local immune response and the status of systemic inflammation [14, 15]. Initially, SII was widely used to assess the prognosis of patients with various kinds of cancer [16], including bladder cancer [17], non-small cell lung cancer [18], rectal cancer [19], and gastric cancer [20]. SII was later found to be also effective in predicting tumorigenesis and identifying individuals at high risk of developing cancer [21]. Several studies have demonstrated that SII is a more accurate predictor of malignancy than conventional immunoinflammatory indicators, including lymphocyte/monocyte ratio (LMR), platelet/lymphocyte ratio (PLR), and neutrophil/lymphocyte ratio (NLR) [19, 22–24]. Overall, the SII provides a noninvasive quantitative standard and is superior to traditional metrics. Considering the association between inflammation and MetS, we assumed that high SII levels could reflect the risk of MetS development, but few studies in this field have been done previously.

Though a study processed by Carmen et al. just focused on one of the cellular components of SII, the link between lymphocytes and MetS has been explained by controlling variables [25]. Moreover, several studies using NHANES database have shown a strong association between SII and certain metabolic diseases, such as diabetic nephropathy [26], hepatic steatosis [27], and osteoporosis [28]. Based on the above, we hypothesized that there is a strong association between SII scores and the risk of MetS. Therefore, to determine the relationship between SII levels of participants from NHANES and the MetS with its subcomponents, we propose to control for confounding variables using more rigorous statistical analysis. We aim to identify individuals at high risk of developing MetS by their SII scores and detect MetS patients earlier.

## Materials and methods

### Study design and population

The NHANES database, which is based on cross-sectional research and intended to evaluate the nutritional and health status of the overall U.S. population, was used to compile data on all study participants. NHANES is affiliated with the Centers for Disease Control and Prevention (CDC) and is updated every two years. We collected data from 2011 to 2016 (2011–2012, 2013–2014, and 2015–2016).

**Fig. 1:**
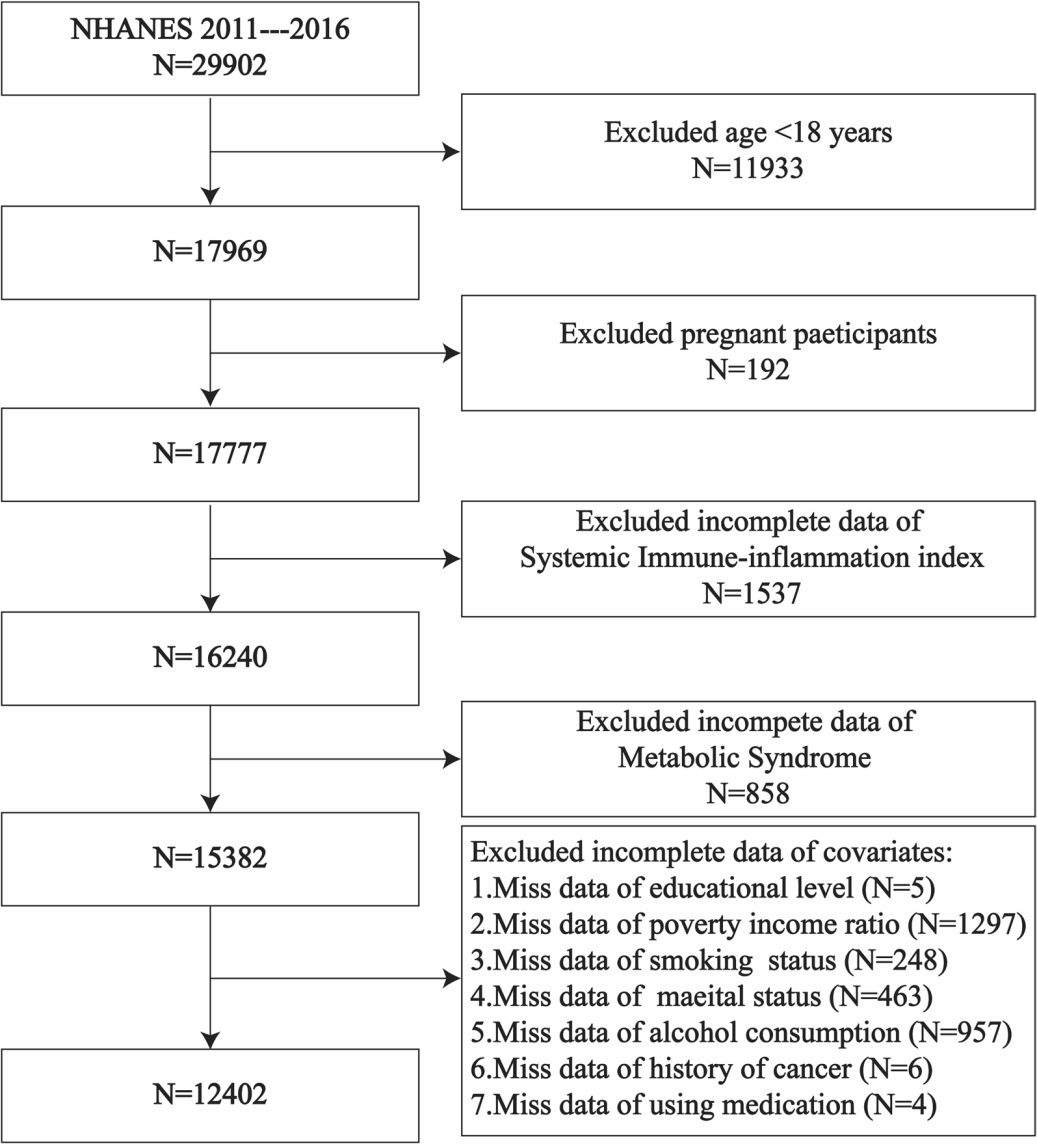
National Health and Nutrition Examination Survey (NHANES) 2011–2016 participant recruitment flow chart

We excluded 11,933 participants younger than 18 years of age and 192 pregnant women, 1,537 participants with incomplete SII data, 858 participants with incomplete MetS data, and 2,980 participants with incomplete concomitant variables (including education level, household poverty income ratio (PIR), smoking status, marital status, alcohol consumption, cancer history, and medication history). The study ultimately included 12,402 participants. The sample selection flowchart is shown in Fig. 1.

### Definition of systemic immunoinflammatory index

Platelet, neutrophil, and lymphocyte counts were assessed by complete blood counts measured using an automated hematology analyzer (Coulter DxH 800 analyzer) and expressed as 1000 cells/**μ**l. As described in prior studies, we estimated SII as platelet count × neutrophil count/lymphocyte count [29]. SII was considered an exposure variable in our analysis.

### MetS definition

MetS is characterized using the diagnostic criteria provided in the Adult Treatment Program III of the National Cholesterol Education Program [30]. The criteria are as follows: (1) TG ≥ 1.69 mmol/L (150 mg/dL); (2) Low HDL-C: < 1.03 mmol/L (40 mg/dL) in men and < 1.29 mmol/L (50 mg/dL) in women; (3) Elevated fasting plasma glucose (FPG) ≥ 6.1 mmol/L (110 mg/dL); (4) Elevated waist circumference (WC): > 102 cm in men and > 88 cm in women; (5) Elevated systolic BP of 130 mmHg and/or elevated diastolic BP of 85 mmHg. Fasting blood samples were obtained during the morning hours after 9 h of fasting, and BP was recorded three times by the physician to establish the mean value.

### Covariates

Participants were provided with standardized questionnaires to collect sociodemographic and lifestyle information. Based on previous studies, we included covariates for adverse cardiometabolic health risk factors, which included low socioeconomic status [31], smoking status [32], alcohol consumption status [32], history of cancer [33], exercise status [34], and family history of diabetes [4]. We included sex (male or female), age continuous or categorical variables (18–39, 40–59, or ≥ 60 years), race/ethnicity (non-Hispanic black, other Hispanic, non-Hispanic white, or other races), marital status categorized as (married, living alone, or divorced), educational attainment (less than high school, high school, and more than high school), PIR (categorized as < 1, 1–2, 2–4, and > 4), and smoking status was categorized as never smoked (< 100 cigarettes before the survey), ever smoked (> 100 cigarettes before the survey but quit before the survey), and current (> 100 cigarettes before the survey and smoked during the survey) as well as self-reported history of cancer, family history of diabetes, and substance use. Participants who drank at least 12 cups of any type of alcoholic beverage (12 oz beer, 5 oz wine, or 1.5 oz liquor) in a year were classified as drinkers. Based on physical activity guidelines that recommend > 75 min of vigorous exercise or > 150 min/week of moderate physical activity, participants were divided into three groups: active (more than the recommended level of activity), less active (less than the recommended level of activity), and inactive (no physical activity).

### Statistical analysis

All statistical analyses were performed per CDC recommendations, using appropriate NHANES sample weights and accounting for the complex multistage subgroup survey. Continuous variables are shown as means with standard errors (SE), and categorical parameters are presented as proportions. Participants were grouped using SII quartiles, and differences between groups were evaluated using weighted linear regression for continuous variables or weighted chi-square testing for categorical variables. Multivariate logistic regression was used to investigate the association between SII and MetS and its components in three distinct models. There was no covariate adjustment in model one. Gender, age, and race-related modifications were made to model two. Model three was adjusted for age, gender, race, education level, PIR, physical activity, smoking status, family history of diabetes, history of cancer, and status of alcohol and substance use. Subgroup analysis of the association between SII and MetS was performed using stratified factors, including sex (male/female), age (18–39, 40–59, or ≥ 60 years), race/ethnicity (non-Hispanic black, other Hispanic, non-Hispanic white, or other races), education (below high school, high school, and above high school), PIR (categorized as < 1, 1–2, 2–4, and > 4), smoking status (never smoked, ever smoked, and currently smoked), and physical activity (active, less active, and inactive). These stratification variables were also considered as pre-specified possible impact modifiers. To test for heterogeneity of associations between subgroups, an interaction term was also introduced.

The nonlinear relationship between SII and MetS and its components was tested using restricted cubic spline (RCS) regression. The likelihood ratio test was used to confirm this relationship. It is worth noting that SII were transformed into logarithms when performing regression analyses because they were right-skewed distributions. R software (version 4.1.2; https://www.R-project.org) was used for all analyses.

## Results

### Baseline characteristics of participants

The sample size of this study was 12,402 participants, including 6,198 males and 6,204 females with a mean age of 47.69 ± 0.72, and 27.18% of the participants were diagnosed with MetS. After grouping the SII quartiles, group Q1 represented the lowest SII group 1.52 ≤ SII < 314.93; group Q2 represented the relatively low SII group 314.88 ≤ SII < 443.78; group Q3 represented the relatively high SII group 443.78 ≤ SII < 626.51; and Q4 represented the highest SII group 626.51 ≤ SII < 8486. The prevalence of MetS in Q1, Q2, Q3, and Q4 groups were 22.70%, 26.11%, 28.32%, and 30.90%, respectively. Among the four SII groups, statistically significant differences were found for age, sex, race, education, marital status, medication history, cancer, diabetes, smoking status, physical activity, FPG, HDL-C, TG, WC, BP, and SII (all P-values < 0.05). Table 1 shows the clinical and biochemical features of patients according to SII quartiles.

**Table 1:** Baseline characteristics of the participants

Variable	No	Q1	Q2	Q3	Q4	P value
Age (years)		46.58 (45.63,47.52)	46.87 (45.96,47.79)	47.46 (46.57,48.35)	49.69 (48.60,50.78)	** < 0.001**
Gender % (SE)						** < 0.001**
Male	6198	54.65 (1.26)	51.84 (1.01)	48.79 (1.32)	43.75 (1.07)	
Female	6204	45.35 (1.26)	48.16 (1.01)	51.21 (1.32)	56.25 (1.07)	
Race/Ethnicity % (SE)						** < 0.001**
Non-Hispanic Black	2652	18.41 (1.93)	9.44 (1.11)	7.47 (0.87)	6.90 (0.79)	
Non-Hispanic White	5020	60.10 (2.78)	68.38 (2.24)	70.22 (2.15)	73.14 (2.12)	
Mexican American	1650	8.04 (1.03)	7.88 (1.10)	8.47 (1.24)	7.88 (1.27)	
Others	3080	13.45 (0.97)	14.31 (1.03)	13.83 (1.00)	12.08 (1.08)	
Education levels % (SE)						**0.012**
Less than high school	2575	15.03 (1.32)	13.15 (1.03)	12.54 (1.07)	14.60 (1.14)	
High school diploma	2733	20.77 (1.28)	19.03 (1.04)	22.20 (0.96)	22.15 (1.22)	
More than high school	7094	64.20 (2.08)	67.83 (1.57)	65.26 (1.39)	63.26 (1.80)	
Marital status % (SE)						**0.001**
Married	7299	64.47 (1.17)	65.80 (1.58)	62.99 (1.37)	59.81 (1.31)	
Single or separated	5103	35.53 (1.17)	34.20 (1.58)	37.00 (1.37)	40.19 (1.31)	
Alcohol consumption % (SE)						0.402
No	3410	22.35 (1.34)	21.54 (1.25)	20.48 (1.41)	20.39 (1.07)	
Yes	8992	77.65 (1.34)	78.46 (1.25)	79.52 (1.41)	79.61 (1.07)	
Use of medication % (SE)						** < 0.001**
No	5209	44.88 (1.18)	42.98 (1.24)	39.62 (1.11)	34.93 (1.16)	
Yes	7193	55.12 (1.18)	57.02 (1.24)	60.38 (1.11)	65.07 (1.16)	
History of cancer % (SE)						**0.016**
No	11,257	91.30 (0.76)	89.33 (0.83)	89.86 (0.77)	87.37 (0.79)	
Yes	1145	8.70 (0.76)	10.67 (0.83)	10.14 (0.77)	12.63 (0.79)	
Family history of diabetes % (SE)						**0.029**
No	10,006	83.01 (0.97)	81.22 (1.01)	78.50 (1.02)	79.32 (1.23)	
Yes	2396	16.99 (0.97)	18.78 (1.01)	21.50 (1.02)	20.68 (1.23)	
Poverty income ratio % (SE)						0.93
< 1	2734	14.66 (1.10)	14.82 (1.03)	14.16 (1.15)	15.35 (1.27)	
1 to 2	3308	20.09 (1.31)	20.75 (0.99)	21.01 (1.04)	21.75 (1.19)	
2 to 4	3210	29.09 (1.40)	28.03 (1.36)	27.95 (1.53)	27.51 (1.39)	
>4	3150	36.16 (2.44)	36.39 (2.13)	36.89 (2.11)	35.39 (1.97)	
Smoking status % (SE)						** < 0.001**
Never smoker	6973	58.45 (1.51)	58.08 (1.20)	56.17 (1.27)	50.87 (1.55)	
Former smoker	2937	23.64 (1.36)	23.87 (1.04)	25.17 (0.87)	26.91 (1.33)	
Current smoker	2492	17.91 (1.17)	18.05 (1.08)	18.66 (1.06)	22.22 (1.07)	
Physical activity % (SE)						** < 0.001**
Inactive	3039	18.05 (1.24)	19.57 (0.94)	20.28 (1.14)	25.55 (0.99)	
Less active	1737	11.16 (0.83)	12.94 (0.79)	13.26 (0.73)	15.55 (0.87)	
Namely active	7626	70.80 (1.33)	67.50 (1.08)	66.46 (1.25)	58.90 (1.25)	
MetS % (SE)						** < 0.001**
No	8913	77.30 (1.03)	73.89 (1.06)	71.68 (1.16)	69.10 (1.00)	
Yes	3489	22.70 (1.03)	26.11 (1.06)	28.32 (1.16)	30.90 (1.00)	
Elevated FPG % (SE)						**0.01**
No	8619	73.39 (0.99)	73.30 (1.19)	72.51 (1.11)	69.20 (1.08)	
Yes	3783	26.61 (0.99)	26.70 (1.19)	27.49 (1.11)	30.80 (1.08)	
Low HDL-C % (SE)						**0.006**
No	8617	73.44 (1.27)	71.73 (1.27)	70.14 (1.42)	68.09 (1.01)	
Yes	3785	26.56 (1.27)	28.27 (1.27)	29.86 (1.42)	31.91 (1.01)	
Elevated TG % (SE)						**0.023**
No	7874	66.22 (1.60)	63.33 (1.22)	62.13 (1.51)	61.36 (1.12)	
Yes	4528	33.78 (1.60)	36.67 (1.22)	37.87 (1.51)	38.64 (1.12)	
Elevated WC % (SE)						** < 0.001**
No	5315	50.78 (1.74)	44.21 (1.37)	38.66 (1.51)	35.64 (1.10)	
Yes	7087	49.23 (1.74)	55.79 (1.37)	61.34 (1.51)	64.36 (1.10)	
Elevated BP % (SE)						**0.002**
No	10,347	87.31 (0.83)	86.20 (0.91)	83.46 (0.85)	83.29 (0.94)	
Yes	2055	12.69 (0.83)	13.80 (0.91)	16.54 (0.85)	16.71 (0.94)	
SII (1,000 cells/µl)		242.55 (240.18,244.93)	381.07 (379.36,382.78)	525.24 (523.09,527.39)	901.37 (887.48,915.27)	** < 0.001**

### Association between SII and MetS

The results of the logistic regression analysis of the incidence of SII and MetS are shown in Table 2. This relationship was shown to be significant in both our unadjusted crude model (odds ratio (OR) = 1.85; 95% confidence interval (CI), 1.47–2.34; P < 0.001) and the least adjusted model (model one) (OR = 1.65; 95% CI, 1.29–2.11; P < 0.001). There was still a positive link between SII and MetS in the fully adjusted model (model two) (OR = 1.44; 95% CI, 1.12–1.84; P = 0.006). This means that each unit rise in log-SII score is related to a 44% increase in MetS prevalence probability. We further converted SII from a continuous variable to a categorical variable (quartiles) for sensitivity analysis, and participants in the highest SII quartile Q4 group had a statistically significant 33% increased risk of MetS compared to participants in the Q1 group with the lowest SII (OR = 1.33; 95% CI, 1.14–1.55; P < 0.001). Compared to the Q1 group, participants in the Q2 and Q3 groups also showed a higher risk of MetS prevalence, with increased risk values of 16% (OR = 1.16; 95% CI, 1.02–1.33; P = 0.027), and 25% (OR = 1.25; 95% CI, 1.10–1.43; P = 0.002), and all of which were statistically significant.

**Table 2:** Association of SII with Metabolic syndrome (MetS) and its components

	Crude model		Model 1		Model 2	
	OR (95%CI)	P value	OR (95%CI)	P value	OR (95%CI)	P value
MetS						
Continous Lg-SII	1.85 (1.47,2.34)	** < 0.001**	1.65 (1.29,2.11)	** < 0.001**	1.44 (1.12,1.84)	**0.006**
Quartile 1	Ref.		Ref.		Ref.	
Quartile 2	1.20 (1.06,1.36)	**0.005**	1.18 (1.04,1.34)	**0.010**	1.16 (1.02,1.33)	**0.027**
Quartile 3	1.35 (1.19,1.52)	** < 0.001**	1.29 (1.14,1.47)	** < 0.001**	1.25 (1.10,1.43)	**0.002**
Quartile 4	1.52 (1.33,1.74)	** < 0.001**	1.43 (1.23,1.66)	** < 0.001**	1.33 (1.14,1.55)	** < 0.001**
P for trend	** < 0.001**		** < 0.001**		** < 0.001**	
Elevated FPG						
Continous Lg-SII	1.36 (1.08,1.71)	**0.010**	1.28 (1.02,1.60)	**0.034**	1.14 (0.89,1.46)	0.275
Quartile 1	Ref,		Ref,		Ref,	
Quartile 2	1.01 (0.87,1.16)	0.948	1.00 (0.86,1.17)	1.000	0.99 (0.84,1.17)	0.904
Quartile 3	1.05 (0.91,1.21)	0.533	1.04 (0.89,1.21)	0.629	1.01 (0.86,1.19)	0.896
Quartile 4	1.23 (1.07,1.41)	**0.004**	1.19 (1.03,1.38)	**0.023**	1.12 (0.96,1.31)	0.153
P for trend	**0.005**		**0.022**		0.136	
Low HDL-C						
Continous Lg-SII	1.50 (1.18,1.91)	**0.001**	1.43 (1.11,1.84)	**0.007**	1.29 (0.99,1.67)	0.056
Quartile 1	Ref,		Ref,		Ref,	
Quartile 2	1.09 (0.94,1.27)	0.251	1.07 (0.92,1.25)	0.382	1.04 (0.88,1.23)	0.607
Quartile 3	1.18 (1.01,1.38)	**0.043**	1.14 (0.97,1.34)	0.120	1.11 (0.94,1.31)	0.213
Quartile 4	1.30 (1.12,1.50)	** < 0.001**	1.26 (1.08,1.46)	**0.003**	1.17 (1.00,1.38)	**0.047**
P for trend	** < 0.001**		**0.003**		**0.038**	
Elevated TG						
Continous Lg-SII	1.44 (1.15,1.81)	**0.002**	1.28 (1.01,1.63)	**0.044**	1.15 (0.91,1.46)	0.241
Quartile 1	Ref.		Ref.		Ref.	
Quartile 2	1.14 (0.98,1.31)	0.083	1.07 (0.93,1.25)	0.341	1.05 (0.90,1.23)	0.494
Quartile 3	1.20 (1.03,1.39)	**0.021**	1.11 (0.95,1.31)	0.196	1.08 (0.91,1.27)	0.375
Quartile 4	1.24 (1.08,1.42)	**0.003**	1.16 (0.10,1.36)	0.052	1.09 (0.93,1.28)	0.265
P for trend	**0.003**		0.052		0.264	
Elevated WC						
Continous Lg-SII	2.76 (2.12,3.60)	** < 0.001**	2.40 (1.82,3.17)	** < 0.001**	2.17 (1.65,2.87)	** < 0.001**
Quartile 1	Ref.		Ref.		Ref.	
Quartile 2	1.30 (1.13,1.51)	** < 0.001**	1.32 (1.12,1.54)	**0.001**	1.31 (1.11,1.53)	**0.002**
Quartile 3	1.64 (1.40,1.91)	** < 0.001**	1.62 (1.37,1.91)	** < 0.001**	1.56 (1.33,1.83)	** < 0.001**
Quartile 4	1.86 (1.60,2.17)	** < 0.001**	1.69 (1.44,1.99)	** < 0.001**	1.59 (1.35,1.87)	** < 0.001**
P for trend	** < 0.001**		** < 0.001**		** < 0.001**	
Elevated BP						
Continous Lg-SII	1.61 (1.17,2.20)	**0.004**	1.74 (1.28,2.37)	** < 0.001**	1.65 (1.20,2.27)	**0.003**
Quartile 1	Ref.		Ref.		Ref.	
Quartile 2	1.10 (0.89,1.36)	0.360	1.19 (0.96,1.47)	0.105	1.19 (0.96,1.48)	0.110
Quartile 3	1.36 (1.15,1.62)	** < 0.001**	1.48 (1.25,1.76)	** < 0.001**	1.46 (1.24,1.72)	** < 0.001**
Quartile 4	1.38 (1.13,1.69)	**0.003**	1.47 (1.21,1.80)	** < 0.001**	1.44 (1.18,1.75)	**0.001**
P for trend	** < 0.001**		** < 0.001**		** < 0.001**	

Crude Model: There are no covariates were adjusted

Model 1: Age, sex, and race/ethnicity were adjusted

Model 2: Age, sex, race/ethnicity, education levels, poverty income ratio, marital status, use of medication, history of cancer , family history of diabetes, smoking status, physical activity, and alcohol consumption were adjusted

In addition, Table 2 depicts the association between the SII and the five MetS-related biochemical indicators in various models. Using multivariate regression analysis with a complex sampling design, we discovered that SII levels were substantially and positively linked with increased WC and BP levels and reduced HDL-C levels, but not with FPG and TG. In particular, for WC, the risk was increased by 56% and 59% in the Q3 and Q4 groups, respectively, with P-values < 0.001. This suggests that the obese population generally has high levels of inflammation, and this finding has very important clinical implications. For BP, the risk was increased by 46% in the Q3 group (OR = 1.46; 95% CI, 1.24–1.72; P < 0.001) and by 44% in the Q4 group (OR = 1.44; 95% CI, 1.18–1.75; P = 0.001), indicating that patients with hypertension are prone to have immune dysfunction than the normal population. The OR for the SII Q2 group was > 1 (OR = 0.99; 95% CI, 0.84–1.17; P = 0.904) for the FPG metric in model two, suggesting that a lower SII score may be negatively associated with participants' blood glucose levels, but interestingly, this association was not significantly different.

### Subgroup analysis

**Fig. 2:**
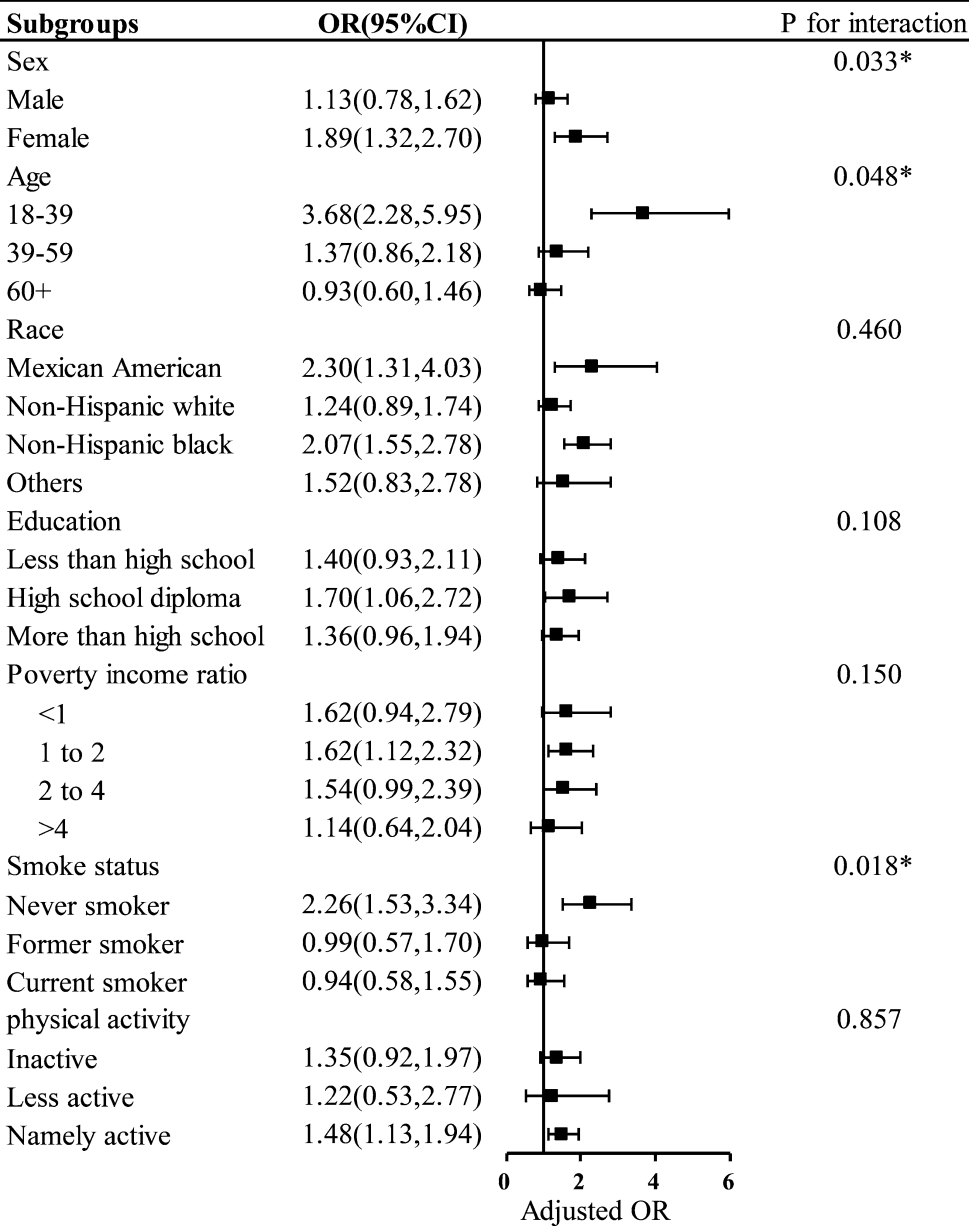
Subgroup analysis of the association between SII and MetS. Odds ratios were calculated based on Log10-SII scores increased by 1. Each stratum was adjusted for age, gender, race/ethnicity, education level, poverty rate, smoking status, and physical activity

Our subgroup analysis revealed that the relationships between SII levels and MetS were not consistent with one another (Fig. 2). Only participants who were female, 18–39 years old, Mexican American, non-Hispanic black, with a high school education background, 1 < PIR < 2, never smoked, and physically active showed statistical significance in subgroups stratified on sex, age, race, education, PIR, smoking status, and physical activity. Although it showed no statistical significance (P > 0.05), SII was positively correlated with MetS in participants who were male, aged 39–59 years, non-Hispanic white people, and of other races. Moreover, we also observed a positive correlation between SII and MetS for participants with a high school education background or above, PIR < 1 or PIR > 2, and those who were physically inactive, but this correlation has no statistical significance. In addition, we also observed a negative association between SII and MetS in those aged > 60 years (OR = 0.93; 95% CI, 0.60–1.46), and in former or current smokers (OR = 0.99; 95% CI, 0.57–1.70); (OR = 0.94; 95% CI, 0.58–1.55.) However, again, this association was not statistically different.

Interaction tests revealed no significant differences in the correlations between SII and MetS for race, education, poverty-to-income ratio, and physical activity, indicating that these factors did not significantly depend on this positive association (all interactions > 0.05). In contrast, gender, age, and smoking status may influence the positive correlation between SII and MetS (interaction P < 0.05).

### Analysis of restricted cubic spline regression

**Fig. 3:**
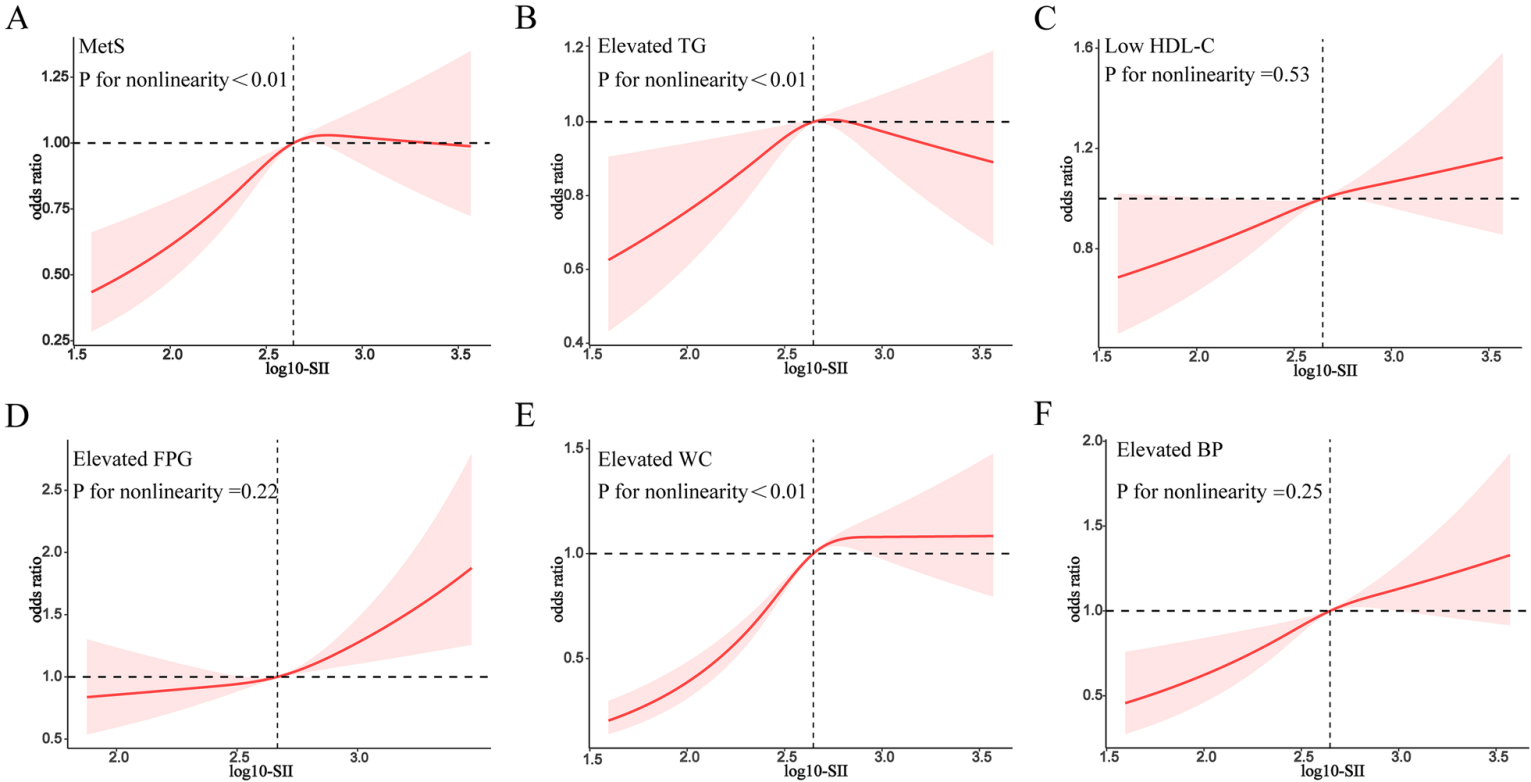
Dose–response relationships between MetS (**A**), Elevated TG (**B**), Low HDL-C (**C**), Elevated FPG (**D**), Elevated WC (**E**), Elevated BP (**F**) and SII. OR odds ratio, CI confidence interval. ORs (solid lines) and 95% confidence levels (shaded areas) were adjusted for age, sex, race/ethnicity, education levels, poverty income ratio, marital status, use of medication, history of cancer, family history of diabetes, smoking status, physical activity, and alcohol consumption. Vertical dotted lines indicate the minimal threshold for the beneficial association with estimated OR = 1

After adjusting for several covariates, we discovered a significant nonlinear connection between SII and MetS in RCS regression (P = 0.012, Fig. 3), with an inverted U-shaped dose–response curve. As shown in Fig. 3, the risk of MetS prevalence tended to increase with log-SII, and this increasing trend gradually slowed down after exceeding 2.65. When log-SII reached 2.83, the OR of MetS showed a decreasing trend as log-SII continued to increase but was located above 1.0.

While examining the specific components of the MetS, the log-SII showed an inverted U-shaped relationship with elevated triglycerides and WC in all participants. Differently, after passing the inflection point, the OR of TG gradually decreased with increasing log-SII and was negatively correlated, whereas the risk of elevated WC gradually leveled off with increasing log-SII. Log-SII was positively correlated with low HDL-C, high FPG, and high BP, although they did not accord with the nonlinear relationship. The nonlinear analysis of MetS and particular components yielded somewhat different results, although the general patterns of the dependent and independent variables were fairly similar in each figure.

## Discussion

As the global economy grows and people's quality of life continues to improve, chronic metabolic diseases, represented by MetS, are becoming the "Top killer" of human health. Many epidemiological studies have demonstrated that immune responses and inflammation are crucial factors in the development of metabolic disorders [35]. To our knowledge, this is the first research that investigates the relationship between SII and MetS in a large, representative sample of adult Americans. In this study, we investigated the linear relationship between MetS and SII using a complicated weighted logistic regression model. The primary outcomes of this study were that SII scores were considerably higher in patients with MetS and that SII levels were positively correlated with the morbidity risk of MetS. This correlation was evident even after controlling for confounding variables, and consistent findings were observed in both continuous and categorical log-SII analyses. Our results provide concrete evidence for further clinical and basic research.

SII is a well-recognized index for predicting cancer treatment efficacy and prognosis. In addition to cancer, the predictive value of SII for other metabolism-related diseases such as CVD is also gaining attention [36, 37]. A Chinese cohort study recruited 13,929 middle-aged and elderly people without heart disease to assess the association between SII and cardiovascular events. Higher SII scores were strongly related to the prevalence of CVD in multivariate Cox regression analysis [38]. To our knowledge, CVD and MetS shared common metabolic pathways [39]. Inspired by this finding, we focused on whether SII has equally value for identification and prediction of MetS.

Recently, an increasing number of studies have started to concentrate on the significance of common indicators in blood routine examination in metabolic disease diagnosis and prevention. Using flow cytometry, Carmen et al. showed that patients with MetS have more lymphocytes than patients without MetS [25]. The increase of patients’ weight has an impact on the physiological state and function of neutrophils [40], and compared to healthy controls, platelet is enhanced in MetS patients. The latter may be associated with epigenetic inheritance [41–43]. Predecessors' conclusions tell us that these cell counts reflecting the level of inflammation are good predictors of MetS. However, most of the traditional inflammatory indicators contain only two types of cells and reflect a relatively limited content. Therefore, SII, which combines three common immune cells, may have greater potential for clinical applications and deserve further exploration in the assessment of MetS incidence.

The main finding of our study was a strong positive association between SII and abdominal obesity and hypertension, as well as a negative correlation with HDL-C. However, SII scores did not show a strong correlation with fasting plasma glucose and serum TG. Animal studies show that in a high-fat diet-induced obesity mice model, macrophages in mouse adipose tissue switch from an M2-polarized to an M1-pro-inflammatory state, while the former could protect adipocytes against inflammatory assault. These phenotypically altered macrophages also simultaneously secrete large amounts of pro-inflammatory adipokines that further exacerbate the systemic inflammatory state [44]. Studies on SII and hypertension are not uncommon, a single-center retrospective study has defined SII as a novel predictor of non-dipper hypertension [45], and a recent study from NHANES noted a U-shaped relationship between all-cause mortality and SII in patients with hypertension [46]. The molecular mechanisms between inflammation and hypertension are relatively complex, with most views suggesting that oxidative stress and vascular endothelial dysfunction are the main causes of elevated BP in a state of systemic inflammation [47].

Interestingly, no association between HDL-C and SII has been previously reported, and we first attempted to bridge SII with HDL-C. HDL-C was considered a protective factor for cardiovascular and other metabolic diseases, and its decreased level causes lipid accumulation in the blood, which in turn induces a severe inflammatory response [48]. This finding is consistent with our observations. The mechanism behind this negative correlation is still unknown, however, the influence of HDL-C on the immune system may take a major role. Results from in vitro cellular assays show that HDL-C reduces the number of activated neutrophils and inhibits neutrophil adhesion and migration [49]. The results of studies based on different populations revealed that HDL-C affects the systemic inflammatory state by altering the levels of NF-κB and TNF-α [50]. Meanwhile, the antioxidant is also one of the important pathways through which HDL-C regulates immunity [51].

In addition to this, we found that the association between SII and serum triglycerides and fasting plasma glucose was not significant, even after adjusting for covariates. Although some studies suggested that hyperglycemia exacerbates the inflammatory response [52], we speculate that the interplay between different immune cells under stress and the presence of selection bias may explain why the above associations were not significant in model 1 and model 2. In a retrospective case–control study, Wang and his colleagues did not detect significant differences in total neutrophil counts between women with gestational diabetes and controls [53]. A cross-sectional survey from Brazil also showed no significant difference in NLR scores between normal and hyperglycemic participants [54], which is consistent with what we have observed so far. Similar reports emerged in an endless stream, and another point we should consider is that changes in patients' poor lifestyle habits due to previously diagnosis like hyperglycemia and hyperlipidemia may have an impact on the observed results.

In this study, subgroup analysis and interaction tests demonstrated that the positive association between SII and MetS was not consistent across subgroups and differed significantly by gender, age, and smoking status. Varying responses to sex hormones may assist to explain the prevalence of gender variations in the SII-MetS relationship [55]. A meta-analysis found that lower levels of sex hormone-binding globulin and greater levels of estradiol raise the likelihood of diabetes in women [56]. A large amount of evidence suggests that hypertension and obesity are also associated with differences in sex hormone levels [57, 58]. It is generally believed that estrogen exacerbates the inflammatory response in individuals [59], which may explain the stronger association between SII and MetS in women. Another possible reason is that male patients with MetS tend to be more frequently exposed to smoking, which could also lead to the sex differences. Our results found a stronger positive correlation between SII scores and MetS in never-smokers as well. Smoking increases the number of neutrophils in the airways of patients with asthma, who show a significantly skewed distribution of SII scores [60]. The immune system in the smoking population is often in a state of disorder, and over-activated levels of inflammation bias the results of the subgroup analysis somewhat, obscuring the true effect of elevated SII levels on the incidence of the MetS. The results of the subgroup analysis also suggest that there are age differences in the relationship between SII and MetS. One report indicated that when an external infection is present, the degree of immune activation is not the same in different age groups and that the higher the age, the weaker the immune response [61]. This may be related to decreased immune cell function and a reduced number of pattern recognition receptors in older populations. The theory of "aging inflammation" was recognized during the COVID-19 epidemic [62]. Many chronic diseases in older individuals, such as coronary artery disease and COPD, also inhibit the immune system from functioning properly [61]. Moreover, we discovered a negative relationship between SII and MetS in patients above the age of 60. However, this was not statistically significant. We hypothesize that this occurrence is due to a inadequate sample size in this age group.

Moreover, the magnitude of association and trend analyses in logistic regression were assessed in this study, and the dose–effect connection was investigated using RCS analysis. The link between SII and MetS was discovered to be an inverted U-shaped RCS curve. A rise in log-SII below the threshold was strongly related to the risk of MetS development. However, above the threshold, the OR of MetS gradually plateaued with a further increase in log-SII dose. Several studies have shown that in organisms, receptor-mediated responses start out showing a strong dependence on increasing doses and cease to be sensitive to increasing doses after reaching a peak [63]. Insulin resistance is the core mechanism linking inflammation and MetS, and saturation of insulin receptors, as an early manifestation and major cause of MetS, might be the main reason for the gradual flattening of the rising curve [64]. Similarly, saturation exists at others, such as TG and leptin receptors. Another possible explanation is that we believe that the smoothing component after the inflection point may be associated with missing data in patients with high SII scores. An inverted U-shaped relationship between log-SII and specific components of MetS, such as abdominal obesity and hypertriglyceridemia, was also observed in this study. However, there was no nonlinear relationship between log-SII and low levels of HDL-C or high levels of FPG or BP, and the mechanism behind this warrants further investigation.

Our study holds several distinct advantages. Firstly, our study is the first to explicitly investigate the role of SII in MetS. Additionally, we analyze the association between SII and each subset of MetS, interpreting the results separately. This approach showcases the rigorous nature of our study. Secondly, our study utilized a cross-sectional investigation of a large, nationally representative sample cohort. This methodology allowed us to comprehensively control for confounding factors. Moreover, the substantial sample size enabled us to conduct subgroup analyses, thereby exploring the potential influence of other factors on the association between SII and MetS. As a result, our findings are more representative and valid, allowing for the generalization of our results to the broader adult population in the United States. Thirdly, we adjusted the SII scores to account for continuous and categorical variables. Furthermore, we separated the analysis of each variable to minimize potential eventualities during statistical analysis, enhancing the reliability of our findings.

However, there are some limitations to this study. First, due to the initial design of the NHANES database, we were only able to measure platelet, neutrophil, and lymphocyte counts at a single time point at baseline status, but these counts may have changed over time with follow-up. Moreover, in the actual analyses work, we tended to exclude participants who had incomplete data like missing cell counts, which leaded to the appearance of selection bias. Although we have adjusted the data to the greatest extent possible, it is undeniable that this bias still exists. Hence, our study's findings may not accurately represent all individuals. Second, the cross-sectional investigation hindered us from defining the causal relationship and temporal order between SII and MetS. Thirdly, despite adjusting for a large number of possible confounders, we were unable to remove fully the impact of unmeasured confounders. Therefore, we should be prudent about our conclusions, which require us to conduct more detailed studies on groups with different diseases in the future.

Despite some limitations and shortcomings, our study still has tremendous clinical significance. As a novel non-invasive biomarker of inflammation, SII allows for a more comprehensive way to assess immune and inflammatory responses [65]. This study also confirms our previous conjecture and patients' SII scores will serve as an important basis for the diagnosis of MetS. Especially in modern society, MetS often affects a large portion of the population in grassroots communities. And the SII scores, including three simple and effective hematology indicators, may serve as a straightforward and efficient indicator for primary care physicians to evaluate MetS. In the future, we anticipate conducting a multicenter prospective cohort study to investigate the potential of SII as an independent predictor of MetS. With routine testing of cell counts in patients' blood, our aim is to offer guidance, prevention, and protection to high-risk individuals prior to the onset of metabolic syndrome.

## Conclusion

Our findings suggest a strong association between elevated SII levels and the presence of MetS, particularly in relation to abdominal obesity, hypertension, and HDL-C. Our data indicate that SII shows promise as a straightforward and cost-effective approach to identify individuals with MetS, utilizing the NHANES database and employing more rigorous statistical analysis to account for confounding variables. However, further verification of our findings necessitates larger-scale, multicenter prospective cohort studies.

## Data Availability

The National Health and Nutrition Examination Survey dataset is publicly available at the National Center for Health Statistics of the Center for Disease Control and Prevention (https://www.cdc.gov/nchs/nhanes/index.htm).
